# The Plasma Membrane Calcium Pump in Pancreatic Cancer Cells Exhibiting the Warburg Effect Relies on Glycolytic ATP*

**DOI:** 10.1074/jbc.M115.668707

**Published:** 2015-08-20

**Authors:** Andrew D. James, Waseema Patel, Zohra Butt, Magretta Adiamah, Raga Dakhel, Ayse Latif, Carolina Uggenti, Eileithyia Swanton, Hiromi Imamura, Ajith K. Siriwardena, Jason I. E. Bruce

**Affiliations:** From the ‡ Faculty of Life Sciences,; § Manchester Pharmacy School, and; the ¶ Faculty of Medical and Human Sciences, University of Manchester, Manchester M13 9PT, United Kingdom,; the ‖ Hakubi Center for Advanced Research and Graduate School of Biostudies, Kyoto University, Kyoto 606-8501, Japan, and; the ** Hepatobiliary Surgery Unit, Manchester Royal Infirmary, Manchester M13 9NT, United Kingdom

**Keywords:** ATP, calcium, calcium ATPase, glycolysis, metabolism, pancreatic cancer, Warburg effect, PMCA, calcium overload, calcium pump

## Abstract

Evidence suggests that the plasma membrane Ca^2+^-ATPase (PMCA), which is critical for maintaining a low intracellular Ca^2+^ concentration ([Ca^2+^]*_i_*), utilizes glycolytically derived ATP in pancreatic ductal adenocarcinoma (PDAC) and that inhibition of glycolysis in PDAC cell lines results in ATP depletion, PMCA inhibition, and an irreversible [Ca^2+^]*_i_* overload. We explored whether this is a specific weakness of highly glycolytic PDAC by shifting PDAC cell (MIA PaCa-2 and PANC-1) metabolism from a highly glycolytic phenotype toward mitochondrial metabolism and assessing the effects of mitochondrial *versus* glycolytic inhibitors on ATP depletion, PMCA inhibition, and [Ca^2+^]*_i_* overload. The highly glycolytic phenotype of these cells was first reversed by depriving MIA PaCa-2 and PANC-1 cells of glucose and supplementing with α-ketoisocaproate or galactose. These culture conditions resulted in a significant decrease in both glycolytic flux and proliferation rate, and conferred resistance to ATP depletion by glycolytic inhibition while sensitizing cells to mitochondrial inhibition. Moreover, in direct contrast to cells exhibiting a high glycolytic rate, glycolytic inhibition had no effect on PMCA activity and resting [Ca^2+^]*_i_* in α-ketoisocaproate- and galactose-cultured cells, suggesting that the glycolytic dependence of the PMCA is a specific vulnerability of PDAC cells exhibiting the Warburg phenotype.

## Teaser

**Background:** Pancreatic cancer cells exhibit up-regulated glycolysis (the “Warburg effect”).

**Results:** Reversing the Warburg phenotype protects pancreatic cancer cells from glycolytic inhibitor-induced ATP depletion, plasma membrane calcium pump (PMCA) inhibition, and [Ca^2+^]*_i_* overload.

**Conclusion:** Glycolytic ATP is critical for PMCA function in pancreatic cancer.

**Significance:** The glycolytic dependence of the PMCA may represent a novel therapeutic target in pancreatic cancer.

## Introduction

Pancreatic ductal adenocarcinoma (PDAC)^5^ remains a significant unsolved global health problem. The 5-year survival rate remains below 5% (1), representing an almost entirely unmet clinical need for effective treatment options. Recent years have seen increased focus on the role of altered metabolism in numerous cancers (2), including PDAC (3), with the aim of identifying novel targets. A common hallmark of cancer is an aberrant metabolic profile characterized by a high glycolytic rate, despite the abundance of O_2_ (the “Warburg effect”). This phenomenon, which occurs in PDAC (4, 5), is thought to confer numerous tumor survival advantages, including resistance to hypoxia and the generation of glycolytic intermediates for macromolecule biosynthesis (6). Nevertheless, despite this metabolic reprogramming, a robust production of ATP remains key for fueling critical energy-dependent processes within tumor cells, particularly in the face of hypoxia (7, 8).

One such process is Ca^2+^ efflux via the ATP-dependent plasma membrane calcium (Ca^2+^)-ATPase (PMCA), which maintains a low resting cytosolic Ca^2+^ concentration ([Ca^2+^]*_i_*, ∼100 nm). The PMCA is the predominant Ca^2+^ efflux pathway in human PDAC cells (9), and its inhibition results in an irreversible increase in [Ca^2+^]*_i_* (“[Ca^2+^]*_i_* overload”) and cell death (10). The PMCA therefore has a crucial role in [Ca^2+^]*_i_* homeostasis and cell survival. We have previously reported that the PMCA in PDAC utilizes glycolytically derived ATP and that glycolytic inhibition resulted in profound ATP depletion, PMCA inhibition, [Ca^2+^]*_i_* overload, and cell death (9). We speculated that this may present a cancer-specific weakness; however, it is unknown whether the glycolytic dependence of the PMCA also occurs in healthy cells more reliant on mitochondrial metabolism.

To examine this, this study sought to reverse the highly glycolytic phenotype of PDAC cells and to determine the importance of the relative source of ATP (mitochondrial *versus* glycolytic metabolism) for fueling the PMCA. Evidence indicates that glucose deprivation from culture medium, while supplementing with substrates that promote mitochondrial metabolism, represents an *in vitro* model of aerobically poised noncancerous cells *in vivo* (11). Thus, in this study, glucose-deprived PDAC cells were supplemented with one of two substrates reported to promote mitochondrial metabolism as follows: the monosaccharide sugar galactose or the keto-analogue of leucine, α-ketoisocaproate (KIC).

Galactose is converted via the Leloir pathway to glucose 6-phosphate, thus bypassing hexokinase and entering glycolysis at a slower rate than glucose (12). Evidence suggests that cell culture in galactose results in an increased reliance on mitochondrial metabolism (11, 13). In contrast to galactose, KIC is metabolized within the mitochondria, enhancing the availability of α-ketoglutarate (14, 15), acetyl-CoA, and the ketone body acetoacetone (16, 17), which can then be utilized to fuel increased mitochondrial respiration (18). Ketone bodies are also thought to contribute to the anticancer effects of the ketogenic diet on PDAC by inducing metabolic reprogramming (19). We therefore hypothesized that KIC and galactose would be good substrates with which to shift the metabolic phenotype of cultured PDAC cells toward mitochondrial metabolism.

We report that a relative shift from glycolytic to mitochondrial metabolism can be achieved in human PDAC cells (MIA PaCa-2 and PANC-1) by culturing in glucose-deprived conditions supplemented with either KIC (2 mm) or galactose (10 mm). This corresponded to a reversal in sensitivity to ATP depletion by inhibitors of either glycolytic or mitochondrial metabolism. Moreover, the previously reported effects of the glycolytic inhibitor iodoacetate (IAA) on [Ca^2+^]*_i_* overload and PMCA activity in highly glycolytic MIA PaCa-2 cells (9) were profoundly attenuated or absent following their culture in KIC and galactose. These results indicate that the PMCA in PDAC relies on glycolytically derived ATP when glycolytic flux is high, which may represent a cancer-specific vulnerability in PDAC cells exhibiting the Warburg phenotype. Therefore, targeting this glycolytic ATP supply to the PMCA may represent a novel therapeutic strategy for the treatment of PDAC.

## Experimental Procedures

### 

#### 

##### Cell Culture

PANC-1 and MIA PaCa-2 cells (ATCC) were cultured in a humidified atmosphere of air/CO_2_ (95:5%) at 37 °C, in either glucose-containing DMEM (D6429, Sigma) or glucose-free DMEM (11966-025, Life Technologies, Inc.) supplemented with 10 mm d-(+)-galactose (galactose, Sigma) or KIC (Sigma). All media were supplemented with 10% FBS, 100 units/ml penicillin, 100 g/ml streptomycin.

##### Cell Proliferation Assay

MIA PaCa-2 cells (5000 cells per well, eight replicates) were fixed at 2, 24, 48, 72, and 96 h post-seeding using 10% trichloroacetic acid (4 °C for 1 h), rinsed with H_2_O, dried, and stained using sulforhodamine B. Excess dye was removed using 1% acetic acid, and the remaining dye was solubilized using a standard volume of 10 mm Tris. Protein content was measured as absorbance at 565 nm (absorbance units, AU). To assess proliferation rate, absorbance between 72 and 96 h (AU/h) was compared using a one-way ANOVA with post hoc Bonferroni's test.

##### Luciferase-based ATP Assays

ATP content of MIA PaCa-2 and PANC-1 cells (seeded overnight at 1 × 10^5^ cells/ml) was determined after metabolic inhibitor treatment using a ViaLight Plus kit (Lonza) and a Synergy HT reader (BioTek). Experiments were run in duplicate. Background luminescence values from a positive control (ATP depletion mixture: 10 μm OM, 4 μm carbonyl cyanide *m*-chlorophenyl hydrazine, 2 mm IAA, and 500 mm BrPy) were subtracted from all values before normalizing to untreated control (%). Comparisons between groups were performed using a Kruskal-Wallis test with a post hoc Dunn's test.

##### Extracellular Flux Measurements

XF mito stress or XF glycolysis stress tests were performed using an XFe96 Analyzer (Seahorse Bioscience) according to the manufacturer's instructions with optimized drug and substrate concentrations. Glucose-, galactose-, and KIC-cultured MIA PaCa-2 cells were seeded at 2.5 × 10^4^ cells per well. For the XF mito stress test, XF base assay medium was supplemented with either 10 mm glucose and 1 mm sodium pyruvate, 2 mm KIC, or 10 mm galactose as appropriate. Three sequential measurements were recorded at set intervals prior to drug addition (basal) and following each sequential drug addition. Each third measurement was used to calculate the metabolic parameters reported (Figs. 3*B* and 4*B*). Data were normalized using a sulforhodamine B-based protein assay. Replicates from a single experiment (XF mito stress test, 6–8 replicates; XF glycolysis stress test, 14–16 replicates) were averaged to give the presented experimental mean ± S.E. Statistical comparisons were performed using a one-way ANOVA with a post hoc Bonferroni test.

##### Fura-2 Fluorescence and GO-ATeam FRET Imaging

To measure [Ca^2+^]*_i_*, cells were loaded with fura-2 AM (4 μm) prior to mounting on an imaging system comprised of a Nikon Diaphot, a ×40 oil immersion objective (numerical aperture 1.3), and an Orca CCD camera (Hamamatsu), as described previously (9). Excitation light (340 and 380 nm, 50-ms exposure) was separated from emitted light using a 400-nm dichroic with a 505LP filter.

To assess ATP depletion, MIA PaCa-2 cells stably expressing GO-ATeam (20) were generated by transfecting with GeneCellin (BioCellChallenge) followed by selection with G418 (500 μm, Sigma). Cells were mounted onto a Nikon TE2000 microscope fitted with a ×40 oil immersion objective (numerical aperture 1.3) and a CoolSNAP HQ interline progressive-scan CCD camera (Roper Scientific Photometrics). Excitation light (470 nm, 500 ms exposure) was separated from emitted light using a 505-nm dichroic mirror with a dual band emission filter (59004m ET FITC/TRITC Dual Emitter). Emitted light was simultaneously collected at 510 nm and above 560 nm using an OptoSplit Image Splitter and a JC1 565 nm dichroic mirror (Cairn Research).

All cells were perfused at room temperature with HEPES-buffered physiological saline solution (HEPES-PSS: 138 mm NaCl, 4.7 mm KCl, 1.28 mm CaCl_2_, 0.56 mm MgCl_2_, 5.5 mm glucose, 10 mm HEPES, pH 7.4); 5.5 mm glucose was replaced with 2 mm KIC and 10 mm galactose for KIC- and galactose-cultured cells, respectively. Background-subtracted images were acquired every 5 s for each emission (GO-ATeam) or excitation (fura-2) wavelength. Both microscope setups employed a monochromator illumination system (Cairn Research) controlled by MetaFluor image acquisition software (Molecular Devices) and gravity-operated perfusion systems (Harvard Apparatus). All cells in an experiment were averaged to give the presented experimental means ± S.E. (*n* = 3–13).

##### Fluorescence Imaging Data Acquisition, Analysis, and Experimental Design

Relative % ATP (GO-ATeam) during a 20-min drug treatment was determined by first subtracting from all FRET ratio values the plateau reached following treatment with a combination of metabolic inhibitors (representing maximal ATP depletion). These values were then normalized to pretreatment baseline (% ATP) and assessed using a two-way ANOVA with post hoc Bonferroni test.

Resting [Ca^2+^]*_i_* was estimated by plotting calibrated fura-2 ratios from MIA PaCa-2 cells (three experiments, 97 cells) against log[Ca^2+^]*_i_*, and the resulting curve (representative of an average cell) was extrapolated to calculate [Ca^2+^]*_i_* in all cells assayed with identical imaging settings. Changes in resting [Ca^2+^]*_i_* were measured as the baseline-corrected area under the curve (AUC) and maximum change in [Ca^2+^]*_i_* (max-Δ[Ca^2+^]*_i_*) during 20 min of treatment and assessed using a one-way ANOVA with post hoc Bonferroni's test. Cell viability (% responding to 100 μm ATP with >100 nm increase in [Ca^2+^]*_i_*) was assessed using a Kruskal-Wallis test with a post hoc Dunn's test.

[Ca^2+^]*_i_* clearance was measured using an *in situ* [Ca^2+^]*_i_* clearance assay as described previously (9). Cells were treated with cyclopiazonic acid (30 μm) in Ca^2+^-free HEPES-PSS with 1 mm EGTA, followed by induction of a high [Ca^2+^]*_i_* (using 20 mm Ca^2+^ HEPES-PSS) and its maintenance for 5 min by applying La^3+^ (1 mm) in nominal Ca^2+^-free HEPES-PSS. Cells were then perfused with Ca^2+^-free HEPES-PSS with 1 mm EGTA to allow [Ca^2+^]*_i_* clearance. This was repeated in the presence of metabolic inhibitors. To quantify [Ca^2+^]*_i_* clearance rate and relative recovery, linear rate (60 s) and recovery of [Ca^2+^]*_i_* during the second clearance phase were normalized (%) to that of the first, beginning at the same fura-2 ratio value. Differences between groups were statistically assessed using a Mann-Whitney *U* test.

## Results

### 

#### 

##### Effect of Metabolic Inhibitors on ATP in Galactose- and KIC-cultured PDAC Cells

To shift the metabolism of PDAC cells (MIA PaCa-2 and PANC-1) from glycolytic to mitochondrial metabolism, cells were cultured in nominal glucose-free conditions supplemented with either galactose (10 mm) or KIC (2 mm) for a minimum of three passages and 21 days. Although derived from an identical genetic background, we hypothesized that these cells would resemble a noncancerous metabolic phenotype. Thus, the relative importance of glycolytic ATP for regulation of the PMCA in PDAC could be further corroborated, whereas the original glucose-cultured cells were used as a control in parallel experiments. Glucose-cultured cells were routinely cultured in 25 mm glucose (as recommended by the ATCC for optimal growth and to maintain their highly glycolytic phenotype); however, a more physiological glucose concentration (5.5 mm) was selected during our medium-free imaging experiments to better reflect those conditions present *in vivo*.

**FIGURE 1.**
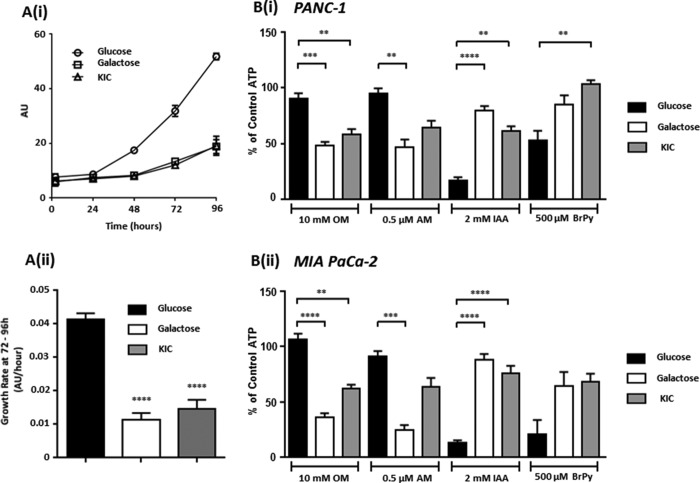
**Culture in KIC or galactose slows growth rate in PDAC cells and switches their sensitivity to metabolic inhibitor-induced ATP depletion.** PANC-1 and MIA PaCa-2 cells were cultured in medium containing glucose (25 mm) or nominal glucose-free DMEM containing either galactose (10 mm) or KIC (2 mm) for a minimum of three passages and 21 days. A sulforhodamine B-based assay for protein content (*AU*, absorbance units) was used to measure proliferation of MIA PaCa-2 cells under these conditions (*A*(*i*), *n* = 8), and their growth rates between 72 and 96 h post-seeding were compared using a one-way ANOVA with post hoc Bonferroni's test (*A*(*ii*)). ATP content of these PANC-1 (*B*(*i*)) and MIA PaCa-2 (*B*(*ii*)) cells in response to 15 min of treatment with mitochondrial (OM, 10 μm; AM, 0.5 μm) or glycolytic inhibitors (IAA, 2 mm; BrPy, 500 μm) was assessed using a luciferase-based assay. Luminescence counts were normalized to untreated control cells (% of control ATP), and statistical comparisons between groups performed using a Kruskal-Wallis test with post hoc Dunn's test, **, *p* < 0.01; ***, *p* < 0.001; ****, *p* < 0.0001, *n* = 4–16.

We first sought to characterize the effects of culturing in KIC, galactose, or glucose on proliferation and metabolism, before subsequently testing the effects of metabolic inhibitors on cytosolic ATP, resting [Ca^2+^]*_i_*, and PMCA activity. Because a common characteristic of highly glycolytic cells is a rapid proliferation, we assessed the proliferation rate of glucose-, galactose-, or KIC-cultured MIA PaCa-2 cells using a sulforhodamine B-based assay. Cell mass was determined at 24-h time intervals (Fig. 1*A*(*i*)), and average growth rates were determined between 72 and 96 h (*n* = 8, Fig. 1*A*(*ii*)). Compared with glucose-cultured cells, the cell proliferation rate between 72 and 96 h was significantly slower in both galactose- and KIC-cultured cells, providing the first indication that these conditions had altered metabolism.

We have previously shown that inhibition of glycolysis, but not mitochondrial metabolism, induces ATP depletion in glucose-cultured PDAC cells (9). To test whether culture in galactose or KIC altered the relative source of ATP production, PANC-1 and MIA PaCa-2 cells from each culture condition were treated with inhibitors of either glycolytic or mitochondrial metabolism for 15 min, and ATP depletion was assessed using a luciferase-based assay. A positive control was performed using a combination of metabolic inhibitors to induce maximal ATP depletion (10 μm OM, 4 μm carbonyl cyanide *m*-chlorophenyl hydrazine, 2 mm IAA, and 500 mm BrPy), with the resulting values subtracted from all other values before normalization to untreated control cells (%).

Treatment with the mitochondrial inhibitors oligomycin (OM, 10 μm) and antimycin A (AM, 0.5 μm) caused a significantly greater ATP depletion in both KIC- and galactose-cultured PANC-1 (Fig. 1*B*(*i*)) and MIA PaCa-2 cells (Fig. 1*B*(*ii*)) than in corresponding glucose-cultured cells. In contrast, the glycolytic inhibitors IAA (2 mm) and 3-bromopyruvate (BrPy, 500 μm) were less effective at depleting ATP in cells cultured in galactose or KIC. This reversal in sensitivity to mitochondrial *versus* glycolytic inhibitors suggests that a significantly higher relative proportion of ATP is derived from the mitochondria in the KIC- and galactose-cultured PDAC cells.

##### Real time Imaging of Cytosolic ATP Using a FRET-based Reporter (GO-ATeam)

To investigate the temporal effects of metabolic inhibitors on ATP depletion in KIC- and galactose-cultured cells, MIA PaCa-2 cells stably expressing the recombinant FRET-based ATP reporter GO-ATeam (20) were cultured under the conditions described above. ATP was then measured by FRET microscopy during 20 min of metabolic inhibitor treatment. This was followed by a combination treatment (IAA, 2 mm; OM, 10 μm; AM, 0.5 μm; 2-deoxyglucose, 10 mm) to induce maximum ATP depletion. The resulting plateau was subtracted from all FRET ratio values, and those at 0, 5, 10, 15, and 20 min were then normalized to the pretreatment baseline (% ATP).

**FIGURE 2.**
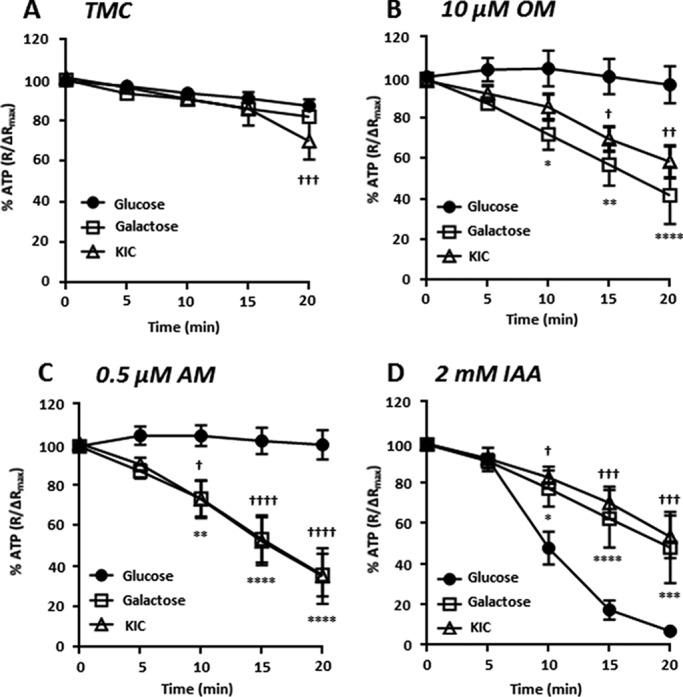
**GO-ATeam FRET imaging reveals the temporal effects of glycolytic and mitochondrial inhibitors on ATP in glucose, galactose-, and KIC-cultured cells.** MIA PaCa-2 cells were cultured in standard Dulbecco's modified Eagle's medium (*DMEM*) containing glucose (25 mm, *closed circles*) or nominal glucose-free DMEM containing either galactose (10 mm, *open squares*) or α-ketoisocaproate (2 mm, *open triangles*) for a minimum of three passages and 21 days. Cytosolic ATP was then measured using GO-ATeam FRET imaging in response to a 20-min treatment with vehicle alone (time-matched control, *TMC*, *A*), OM (10 μm, *B*), AM (0.5 μm, *C*) or IAA (2 mm, *D*). Maximal ATP depletion was induced using a combination of OM, AM, and IAA at equivalent concentrations and 2-deoxyglucose (10 mm). The resulting plateau was subtracted from all FRET ratio values, which were then normalized (%) to the pretreatment baseline (ΔR_max_). Data presented are mean ± S.E. Statistical comparisons were made using a two-way ANOVA with post hoc Bonferroni test at 0, 5, 10, 15, and 20 min treatment. * and † denote significance for galactose- and KIC-cultured cells, respectively, compared with glucose-cultured cells. *, *p* < 0.05; **, *p* < 0.01; ***, *p* < 0.001; ****, *p* < 0.0001, *n* = 3–13 separate experiments.

In control experiments, no difference in ATP was observed over the first 15 min between MIA PaCa-2 cells cultured in galactose or KIC and those cultured in glucose (Fig. 2*A*). However, at 20 min ATP was slightly lower in KIC-cultured cells compared with glucose-cultured cells. Nevertheless, culture in galactose- or KIC-sensitized cells to ATP depletion by OM (10 μm, Fig. 2*B*) and AM (0.5 μm, Fig. 2*C*) was compared with glucose-cultured cells. However, in contrast to the potentiated effects of these mitochondrial inhibitors, the IAA-induced ATP depletion observed in glucose-cultured cells was markedly attenuated in KIC- and galactose-cultured cells (Fig. 2*D*). Together with the data obtained from the luciferase-based assays, this reversal in sensitivity to ATP depletion by glycolytic and mitochondrial inhibitors suggests that a shift from glycolytic to mitochondrial metabolism can be achieved using glucose-deprived medium containing either galactose or KIC.

##### Mitochondrial and Glycolytic Metabolism Are Altered in Galactose- or KIC-cultured Cells

Because culture in KIC and galactose resulted in a switch in sensitivity to metabolic inhibitors with respect to ATP depletion, we assessed the metabolic profile of MIA PaCa-2 cells cultured in these conditions using a Seahorse Bioscience XFe96 analyzer. The XFe96 simultaneously measures both O_2_ consumption rate (OCR) and extracellular acidification rate (ECAR) of cells, providing a temporal measure of mitochondrial and glycolytic function in response to test reagents.

**FIGURE 3.**
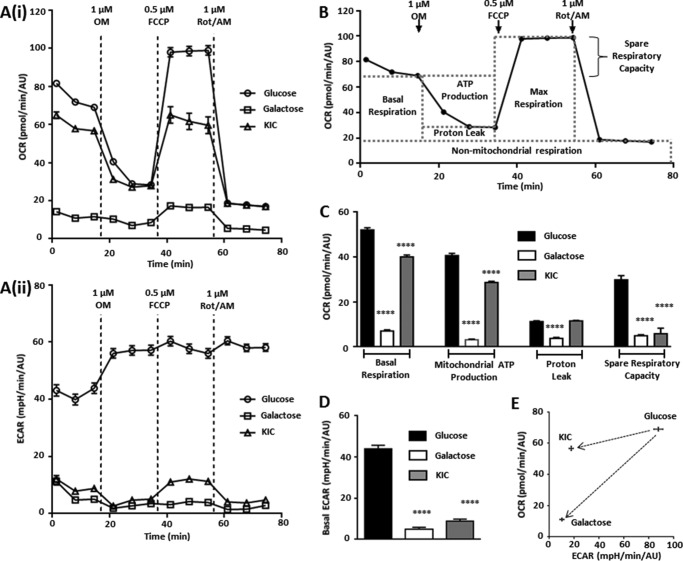
**MIA PaCa-2 cells cultured in galactose or KIC exhibit alterations in mitochondrial and glycolytic metabolism compared with glucose-cultured cells.** After culturing MIA PaCa-2 cells in media containing glucose (25 mm), galactose (10 mm), or KIC (2 mm) for 21 days and three passages, mitochondrial function was assessed using a Seahorse XFe96 Analyzer and an XF mito stress test. Sequential addition of OM (1 μm), carbonyl cyanide *p*-trifluoromethoxyphenylhydrazone (*FCCP*) (0.5 μm), and a combination of rotenone and antimycin A (*Rot/AM*, both 1 μm) revealed the components of mitochondrial metabolism. OCR (*A(i*)) and ECAR (*A(ii*)) were measured in glucose-cultured (*open circles*), galactose-cultured (*open squares*), and KIC-cultured (*open triangles*) cells, and all measurements were normalized using a sulforhodamine B assay for protein content (AU, absorbance units). *B*, XF mito stress test and measurement of mitochondrial parameters, calculated using every third measurement. Mitochondrial parameter values (*C*) and basal ECAR values (*D*) in glucose (*n* = 15) galactose (*n* = 16) and KIC (*n* = 14) cultured cells are presented (mean ± S.E.). Statistical comparisons were performed using a one-way ANOVA with post hoc Bonferroni's test for multiple comparisons, ****, *p* < 0.0001. A basal OCR *versus* basal ECAR plot (*E*) for glucose- (*n* = 45), galactose- (*n* = 48) and KIC-cultured cells (*n* = 42) indicated the shift in basal metabolism for KIC- and galactose-cultured cells.

The mitochondrial function of MIA PaCa-2 cells was first assessed using an XF mito stress test (Fig. 3, *A*(*i* and *ii*) and *B*). Surprisingly, both galactose- and KIC-cultured cells exhibited a significantly lower basal respiration OCR, ATP production-linked OCR, and spare respiratory capacity compared with glucose-cultured cells (Fig. 3, *A*(*i*) and *C*), indicating that despite their highly glycolytic phenotype, MIA PaCa-2 cells retain functional mitochondria. Nevertheless, despite an apparent decrease in mitochondrial function, basal ECAR in galactose- and KIC-cultured cells was significantly attenuated compared with glucose-cultured cells (Fig. 3*D*). An ECAR *versus* OCR plot (Fig. 3*E*) revealed that the basal metabolic phenotype of MIA PaCa-2 cells following culture in galactose or KIC had indeed shifted from a highly glycolytic phenotype to one exhibiting slowed glycolysis, despite a diminished OCR. KIC-cultured cells maintained a relatively high basal OCR despite exhibiting a dramatically decreased ECAR, whereas galactose-cultured cells exhibited a significantly lowered overall metabolism (OCR and ECAR).

Interestingly, blockade of mitochondrial ATP synthesis with OM (1 μm) in glucose-cultured cells resulted in a corresponding increase in ECAR, presumably to maintain ATP (Fig. 3*A*(*ii*)). Crucially, this was absent from galactose- or KIC-cultured cells; these cells could not compensate for the inhibition of mitochondrial ATP production, indicating that they had become more reliant on mitochondrial metabolism. However, we did not know whether this absent compensatory increase in ECAR could be solely attributed to glucose deprivation or whether a functional change in glycolytic capacity contributed.

**FIGURE 4.**
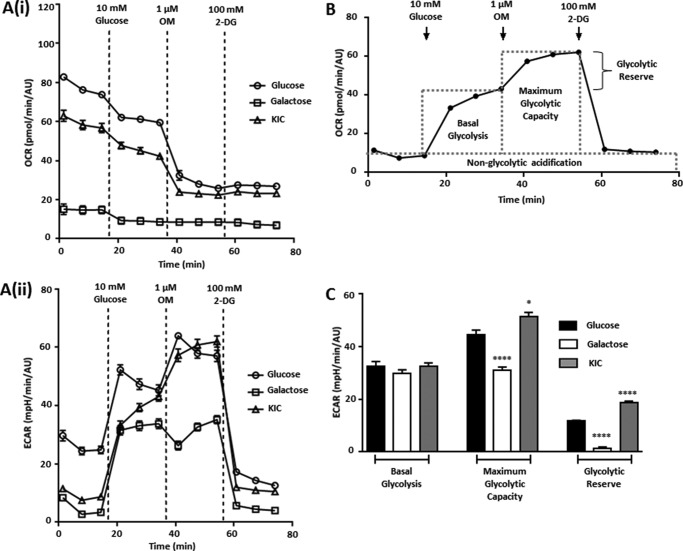
**MIA PaCa-2 cells cultured in galactose or KIC exhibit functional glycolysis upon readdition of glucose.** After culturing MIA PaCa-2 cells in either glucose (25 mm), galactose (10 mm Gal), or KIC (2 mm) for 21 days and three passages, glycolytic function was assessed using an XFe96 analyzer and an XF glycolysis stress test. Sequential addition of glucose (10 mm), OM (1 μm), and 2-deoxyglucose (*2-DG*, 100 mm) revealed the components of glycolytic metabolism. OCR (*A(i*)) and ECAR (*A(ii*)) were measured in glucose- (*open circles*), galactose- (*open squares*), and KIC-cultured (*open triangles*) cells, and all measurements were normalized to protein content using a sulforhodamine B assay for protein content (AU). *B*, XF glycolysis stress test and measurement of glycolytic parameters, calculated using every third measurement. Glycolytic parameter values (*C*) in glucose-cultured (*n* = 7) galactose-cultured (*n* = 8), and KIC-cultured cells (*n* = 6) are presented (mean ± S.E.). Statistical comparisons were performed using a one-way ANOVA with post hoc Bonferroni's test. *, *p* < 0.05; ****, *p* < 0.0001.

We therefore performed an XF glycolysis stress test (Fig. 4, *A*(*i* and *ii*) and *B*) to assess the ability of galactose- or KIC-cultured MIA PaCa-2 cells to utilize glucose as a glycolytic substrate. After 2 h of deprivation of glucose, galactose, or KIC, readdition of glucose (10 mm) induced a sharp increase in ECAR in all three conditions, representing basal glycolysis (Fig. 4*A*(*ii*)). No significant difference in basal glycolysis was observed between glucose-cultured cells and either galactose- or KIC-cultured cells (Fig. 4*C*). However, although the maximum glycolytic capacity (revealed with 1 μm OM) was significantly reduced in galactose-cultured cells compared with glucose-cultured cells, KIC-cultured cells exhibited a significant increase in maximum glycolytic capacity. This was reflected in the glycolytic reserve capacity, which was significantly increased in KIC-cultured cells and decreased in galactose-cultured cells compared with glucose-cultured cells. Thus, despite exhibiting a decreased ECAR in the XF mito stress test, galactose- and KIC-cultured cells largely retained their ability to utilize glycolysis upon glucose exposure, although their maximal glycolytic capacity was affected. Nevertheless, the absence of a reserve glycolytic capacity in the galactose cells suggests that these cells cannot utilize glycolysis as efficiently as glucose-cultured cells. Importantly, when taken together with the previous ATP assays, these results indicate that a switch from a highly glycolytic phenotype to one more reliant on mitochondrial metabolism can be achieved by culturing PDAC cells under these conditions.

##### Culture of MIA PaCa-2 Cells in Galactose or KIC Attenuates the Effects of IAA on Resting [Ca^2+^]_i_

We have previously shown that glucose-cultured PDAC cells exhibit an irreversible [Ca^2+^]*_i_* overload in response to glycolytic inhibitors, although mitochondrial inhibitors had no effect (9), and speculated that this was due to ATP depletion. Because culture in galactose or KIC resulted in a relative shift in metabolism in PDAC cells and a reversal in sensitivity to ATP depletion by metabolic inhibitors, we hypothesized that a corresponding reversal would be observed with regard to [Ca^2+^]*_i_* overload. We therefore assessed the effects of glycolytic *versus* mitochondrial inhibitors on resting [Ca^2+^]*_i_* in these cells over 20 min by measuring area under the curve (AUC) and maximum increase in [Ca^2+^]*_i_* (max-Δ[Ca^2+^]*_i_*) using fura-2 fluorescence imaging.

**FIGURE 5.**
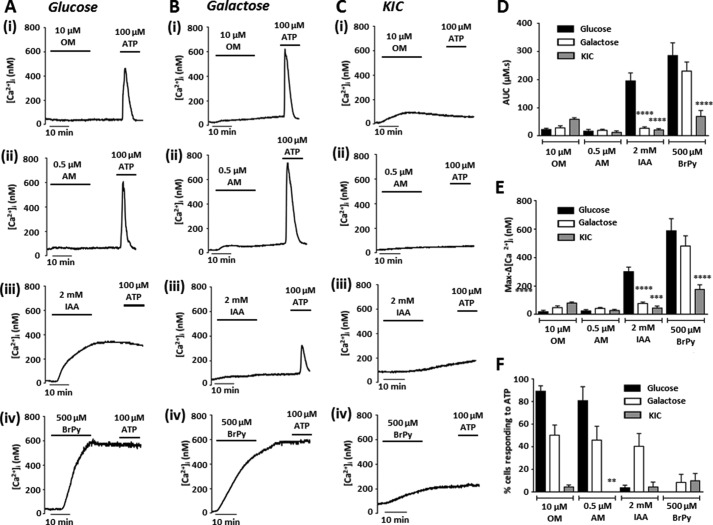
**Culture of MIA PaCa-2 cells in galactose or KIC attenuates the effects of IAA on resting [Ca^2+^]*_i_*.** MIA PaCa-2 cells were cultured in glucose-containing (25 mm) or nominal glucose-free media supplemented with either galactose (10 mm) or KIC (2 mm) for a minimum of three passages and 21 days. Resting [Ca^2+^]*_i_* was then measured in response to 20 min of treatment with metabolic inhibitors using fura-2 fluorescence imaging. Treatment was followed by a 15-min washout and application of 100 μm ATP to test for cell viability. Representative traces from glucose-cultured (*A*), galactose-cultured (*B*), and KIC-cultured (*C*) cells show the effects of OM (10 μm (*i*)), AM (0.5 μm (*ii*)), IAA (2 mm (*iii*)), and BrPy (500 μm (*iv*)) on resting [Ca^2+^]*_i_*. Changes in resting [Ca^2+^]*_i_* were quantified as AUC (*D*) during drug treatment and the maximum change in [Ca^2+^]*_i_* during this period (Max-Δ[Ca^2+^]*_i_*, *E*). Cell viability was assessed by measuring the % cells responding to ATP (Δ[Ca^2+^]*_i_* >100 nm, *F*). Data presented are mean ± S.E., *n* = 4–12 separate experiments. Statistical comparisons were made to glucose-cultured cells. *D* and *E*, one-way ANOVA with post hoc Bonferroni's test; *F*, Kruskal-Wallis test with post hoc Dunn's test, **, *p* < 0.01; ***, *p* < 0.001; ****, *p* < 0.0001.

Following OM or AM treatment, no differences in AUC (Fig. 5*D*) or max-Δ[Ca^2+^]*_i_* (Fig. 5*E*) were observed between either galactose-cultured (Fig. 5*B*(*i* and *ii*)) or KIC-cultured MIA PaCa-2 cells (Fig. 5*C*(*i* and *ii*)) and glucose-cultured control (Fig. 5*A*(*i* and *ii*)). Nevertheless, responsiveness to 100 μm ATP (Δ[Ca^2+^]*_i_* >100 nm), a test for cell viability, was lower in KIC-cultured cells (Fig. 5*F*) than glucose-cultured cells following treatment with AM, indicating that these cells had been sensitized to AM. In contrast, galactose- and KIC-cultured MIA PaCa-2 cells treated with IAA (Fig. 5, *B*(*iii*) and *C*(*iii*)) exhibited dramatically reduced AUC and max-Δ[Ca^2+^]*_i_* responses compared with glucose-cultured cells (Fig. 5*A*(*iii*)). Similarly, the effects of BrPy on AUC and max-Δ[Ca^2+^]*_i_* were significantly reduced in KIC-cultured cells (Fig. 5*C*(*iv*)) compared with glucose-cultured cells (Fig. 5*A*(*iv*)) but not in galactose-cultured cells (Fig. 5*B*(*iv*)). Thus, despite mitochondrial inhibitors having no effect on resting [Ca^2+^]*_i_*, a clear reversal was observed in the sensitivity of KIC- and galactose-cultured cells to glycolytic inhibitor-induced [Ca^2+^]*_i_* overload. The dramatically reduced [Ca^2+^]*_i_* overload responses following IAA and BrPy treatment support the hypothesis that ATP derived from glycolysis is crucial for maintenance of a low resting [Ca^2+^]*_i_* in highly glycolytic MIA PaCa-2 cells.

##### IAA-induced Inhibition of the PMCA Is Abolished in Galactose- and KIC-cultured PDAC Cells

Our previous study demonstrated that inhibitors of glycolysis inhibit Ca^2+^ efflux via the PMCA in PDAC cells, providing a mechanism by which they induce [Ca^2+^]*_i_* overload (9). We therefore next aimed to determine whether KIC- or galactose-cultured MIA PaCa-2 cells were less sensitive to glycolytic inhibitor-induced inhibition of the PMCA. To test this, we selected IAA (2 mm), because its effects on ATP and resting [Ca^2+^]*_i_* were profoundly attenuated in both KIC- and galactose-cultured MIA PaCa-2 cells, and we employed an *in situ* [Ca^2+^]*_i_* clearance assay to isolate and measure PMCA activity (9). Briefly, this involves depletion of the endoplasmic reticulum Ca^2+^ store using cyclopiazonic acid (30 μm), followed by induction of a high [Ca^2+^]*_i_* plateau (using 20 mm Ca^2+^ to induce store-operated Ca^2+^ entry) and its maintenance for 5 min by applying La^3+^ (1 mm), and subsequently allowing Ca^2+^ efflux via the PMCA by applying EGTA (1 mm) in the absence of extracellular Ca^2+^. This is repeated in the presence of test reagents, representing a paired experimental design that controls for cell-to-cell variability. Linear clearance rate (60 s) and relative recovery during the second clearance phase are then normalized to that of the first (%, measured from the same starting fura-2 ratio).

**FIGURE 6.**
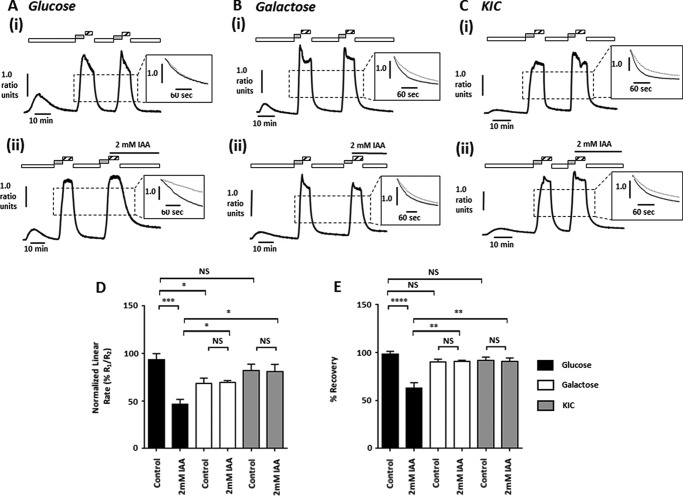
**IAA-induced inhibition of the PMCA is attenuated in galactose- and KIC-cultured PDAC cells.** MIA PaCa-2 cells were cultured in glucose-containing (25 mm) or nominal glucose-free media supplemented with either galactose (10 mm) or KIC (2 mm) for a minimum of three passages and 21 days, and PMCA activity was assessed following glycolytic inhibition with IAA (2 mm). Representative traces show the *in situ* [Ca^2+^]*_i_* clearance assay (PMCA activity) in control (glucose, *A(i*); galactose, *B(i*); and KIC, *C(i*)) and IAA-treated (glucose, *A(ii*); galactose, *B(ii*); and KIC, *C(ii*)) fura-2 loaded MIA PaCa-2 cells. Cyclopiazonic acid (30 μm) was applied in the absence of external Ca^2+^ with 1 mm EGTA (*white box*) or 20 mm Ca^2+^ (*gray box*) to induce store-operated Ca^2+^ influx. 1 mm La^3+^ was applied at the peak of Ca^2+^ influx (*striped box*, 5 min) and subsequently removed with 1 mm EGTA to allow [Ca^2+^]*_i_* clearance. This was repeated in the presence or absence of IAA. *Inset,* expanded time courses are shown comparing the second clearance phase (*gray trace*) with the first (*black trace*). Linear clearance rate (60 s) and relative recovery during the second clearance phase were normalized to that of the first (%), and both were calculated from the same fura-2 ratio value. *D*, mean normalized linear rate (± S.E.). *E*, mean recovery (± S.E.), *n* = 4–9 separate experiments. Statistical comparisons were performed using a Mann-Whitney *U* test, *, *p* < 0.05; **, *p* < 0.01; ***, *p* < 0.001; ****, *p* < 0.0001; *NS*, not significant.

In control galactose-cultured cells (Fig. 6*B*(*i*)), [Ca^2+^]*_i_* clearance rate (Fig. 6*D*) was significantly reduced during the second clearance phase compared with glucose-cultured cells (Fig. 6*A*(*i*)), whereas relative recovery was unaffected (Fig. 6*E*). However, no change was observed in [Ca^2+^]*_i_* clearance rate or relative recovery between untreated KIC-cultured (Fig. 6*C*(*i*)) and glucose-cultured cells. Consistent with previous results (9), IAA profoundly inhibited [Ca^2+^]*_i_* clearance and relative recovery in glucose-cultured cells (Fig. 6*A*(*ii*)), compared with untreated controls (Fig. 6*A*(*i*)). However, IAA had no effect on [Ca^2+^]*_i_* clearance rate or relative recovery in galactose-cultured (Fig. 6*B*(*ii*)) or KIC-cultured cells (Fig. 6*C*(*ii*)) compared with untreated galactose- or KIC-cultured cells (Fig. 6, *B*(*i*) and *C*(*i*)), indicating that glycolytic inhibition with IAA does not affect PMCA activity in these cells. Moreover, relative [Ca^2+^]*_i_* clearance rate and relative recovery were actually significantly higher in IAA-treated galactose- and KIC-cultured cells than in IAA-treated glucose-cultured cells (Fig. 6, *D* and *E*).

These data indicate that galactose and KIC-cultured MIA PaCa-2 cells were effectively resistant to the PMCA inhibition by IAA. In light of the previous results, this is likely due to ATP levels being preserved in the face of glycolytic inhibition due to a shift toward mitochondrial metabolism and a decreased reliance on glycolysis.

## Discussion

We have previously demonstrated that glycolytic inhibition in PDAC cell lines (PANC-1 and MIA PaCa-2) results in ATP depletion and an inability to maintain a low resting [Ca^2+^]*_i_* that is most likely due to inhibition of the PMCA (9). To further interrogate the glycolytic ATP dependence of the PMCA, this study aimed to shift highly glycolytic PDAC cells toward mitochondrial metabolism by culturing in glucose-deprived conditions supplemented with either galactose (10 mm) or KIC (2 mm). Under these conditions, proliferation rate and ECAR were both markedly decreased, indicating a relative reversal of the Warburg phenotype. Moreover, the sensitivity of these cells to metabolic inhibitors was reversed; ATP depletion following glycolytic inhibition (IAA or BrPy) was markedly reduced, whereas the effects of mitochondrial inhibitors (OM and AM) were potentiated. Importantly, these cells became resistant to IAA-induced [Ca^2+^]*_i_* overload and PMCA inhibition. This is in direct contrast to highly glycolytic glucose-cultured cells, which were exquisitely sensitive to an acute challenge by IAA. This apparent reversal in sensitivity to glycolytic inhibition suggests that a glycolytic ATP supply to the PMCA is not important in mitochondrially poised cells with a relatively slow glycolytic flux. Moreover, these findings corroborate the hypothesis that glycolytic ATP is critical for PMCA function in PDAC cells exhibiting a high glycolytic rate.

The luciferase and GO-ATeam-based assays revealed that culturing in galactose or KIC reversed the relative sensitivity of PDAC cells to ATP depletion by mitochondrial and glycolytic inhibitors. As shown previously (9), the glycolytic inhibitors BrPy and IAA both caused dramatic ATP depletion in glucose-cultured PDAC cells. However, this was markedly attenuated in cells cultured in galactose or KIC while the effects of mitochondrial inhibitors were potentiated, indicating a shift toward a greater reliance on mitochondrial metabolism. Similarly, previous studies indicate that cancer cells cultured in galactose medium exhibit increased expression of mitochondrial respiratory chain proteins (13) and an increased reliance on mitochondrial ATP production (11, 21). In this study, this shift in metabolism likely occurs due to a relative decrease in glycolytic flux rather than its complete cessation, because glycolysis is slowed but functional in the presence of galactose (12) and a small amount (∼0.5 mm) of glucose is present in the culture media due to serum supplementation. Nevertheless, the results indicate that PDAC cells exhibit a degree of metabolic adaptability to substrate availability and retain functional mitochondria for ATP production should certain conditions prevail. This is supported by the extracellular flux (XF) assays in this study. Although Warburg initially suggested up-regulated glycolysis in cancer cells stemmed from mitochondrial defects (22), it is now appreciated that mitochondria in cancer cells are indeed functional (23).

In addition to revealing their mitochondrial capacity, the XF assays confirmed the high glycolytic rate of MIA PaCa-2 cells when cultured in glucose. Importantly, however, basal ECAR was markedly decreased following their culture in galactose or KIC media, providing an explanation for the observed reversal in sensitivity to ATP depletion by metabolic inhibitors. Moreover, the slowed proliferation rate in these cells could also be attributed to the decreased ECAR, because attenuated glycolytic flux would likely lower the availability of glycolytic intermediates for use as substrates for cell proliferation. However, although relatively well maintained in KIC-cultured cells, OCR was significantly reduced in galactose-cultured cells, presumably due to the slower rate of galactose entry into glycolysis (12) decreasing overall metabolic rate. In contrast, OCR was maintained in KIC cells, suggesting that KIC is a better substrate for promoting mitochondrial metabolism following glucose deprivation. Importantly, although glucose-cultured cells could compensate for the OM blockade of mitochondrial ATP synthesis by increasing glycolytic flux, as evidenced by an increase in ECAR, this was not observed in galactose- and KIC-cultured cells. This indicates that the KIC- and galactose-cultured cells lack the compensatory glycolytic component that allows glucose-cultured cells to rapidly increase glycolysis and maintain ATP, and this supports the notion that they had become more reliant on mitochondrial ATP production.

Although it is now appreciated that mitochondria can contribute to ATP production in cancer cells, until recently the predominant hypothesis had been that glycolysis provides the bulk of ATP following metabolic transformation. It is now thought that a high glycolytic rate instead supports the production and diversion of glycolytic intermediates to anabolic pathways for cell proliferation (6). However, the large amount of lactate excreted presents a functional paradox, because these are carbons that could instead be incorporated into cell proliferation, and suggests the high glycolytic rate exhibited by cancer may have a bioenergetic component as well as a predominant biosynthetic component (23). This may be particularly pertinent when PDAC cells are faced with low O_2_ availability within a hypoxic tumor core (7, 8). Nevertheless, the degree to which glycolysis contributes to ATP in cancer remains a controversial topic, as the available evidence thus far has been equivocal. Although some studies indicate that cancer cells derive a majority of ATP from mitochondria (7, 24–26), others suggest that ATP is predominantly generated by glycolysis (9, 21, 27–29). However, it is likely that cancer cells exhibit a degree of metabolic adaptability to respond to changes in tumor hypoxia and substrate availability (30, 31). This is supported by this study, where PDAC cells could adapt to survive in glucose-deprived conditions, but in doing so they became sensitive to ATP depletion by mitochondrial inhibitors.

Despite their ability to adapt to long term glucose deprivation and changing substrate availability, glucose-cultured MIA PaCa-2 cells appear unable to rapidly adapt their metabolism following acute glycolytic inhibition to maintain [Ca^2+^]*_i_* homeostasis. As in our previous study (9), IAA induced ATP depletion, PMCA inhibition, and [Ca^2+^]*_i_* overload in glucose-cultured cells, which is well known to induce cell death (10). Crucially, however, the resistance of KIC- and galactose-cultured cells to ATP depletion by glycolytic inhibition conferred resistance to IAA-induced PMCA inhibition and [Ca^2+^]*_i_* overload. These data provide the first evidence that the glycolytic dependence of the PMCA might be specific to cancer cells exhibiting a highly glycolytic phenotype. Moreover, the exquisite sensitivity of highly glycolytic MIA PaCa-2 cells to glycolytic inhibition coupled with the resistance of KIC- and galactose-cultured cells has important implications for the potential selective targeting of [Ca^2+^]*_i_* homeostasis in PDAC. Based on these results, a treatment strategy targeting the glycolytic dependence of the PMCA in PDAC might be expected to leave adjacent healthy cells unaffected; in a similar fashion to KIC- and galactose-cultured cells, noncancerous cells would be expected to rely on mitochondrial metabolism rather than glycolysis for the bulk of ATP production.

An important caveat to targeting the glycolytic dependence of the PMCA in PDAC concerns the possibility of resistance, as some cancer cells in a heterogeneous tumor might be expected to exhibit decreased sensitivity to this therapeutic strategy due to differences in their metabolic phenotype. In particular, those cells on the periphery of a tumor might exhibit a higher reliance on mitochondrial metabolism over glycolysis due to their proximity to increased vasculature (30, 31); in these cells, a less glycolytic phenotype might be expected to confer resistance to the targeting of glycolytic metabolism. However, aggressive tumors are commonly driven by cells exhibiting up-regulated glycolytic metabolism, and this metabolic phenotype is associated with increased proliferation, invasion, and metastasis and a poorer prognosis (32). Thus, targeting the glycolytic dependence of the PMCA as part of a combination treatment strategy might be an effective means of eliminating the more aggressive (highly glycolytic) cells within a tumor cell population, thus leading to an improved treatment outcome.

Although a clear reversal was observed in the sensitivity of KIC- and galactose-cultured cells to [Ca^2+^]*_i_* overload by IAA and BrPy, we were initially concerned by the lack of response of the KIC-cultured cells to ATP, a *bona fide* agonist capable of coupling to Ca^2+^ signaling, as this potentially indicated a loss of cell viability. However, upon closer inspection, KIC cells did appear to respond to ATP, but this response was so severely attenuated that it often did not meet the criteria selected for a successful response to ATP (an increase in [Ca^2+^]*_i_* of 100 nm or greater above baseline) and could not be recorded with confidence. That KIC-cultured cells from the same flasks responded as expected during our *in situ* [Ca^2+^]*_i_* clearance assay and XF assays indicates that these cells were indeed viable. It is instead likely that one or more components of the ATP-induced Ca^2+^-signaling machinery was inhibited following culture in KIC; however, the exact reason for this remains unknown.

Despite the findings that KIC- and galactose-cultured cells are less sensitive to treatment with IAA, not all metabolic inhibitors produced such a clear-cut reversal of responses as observed with IAA. A similar reversal in ATP depletion was observed with BrPy, and yet the BrPy-induced [Ca^2+^]*_i_* overload response remained in galactose-cultured cells. However, BrPy has been reported to dissociate hexokinase from voltage-dependent anion channel (33) and depolarize the mitochondrial membrane potential (ΔΨm (34)), and PMCA inhibition and [Ca^2+^]*_i_* overload have been shown to follow loss of ΔΨm without accompanying ATP depletion (35). BrPy has also been reported to increase oxidative stress (36), which is known to inhibit PMCA activity (35). The effects of BrPy on [Ca^2+^]*_i_* overload are therefore less clear, resulting in our decision to only proceed with IAA to the [Ca^2+^]*_i_* clearance assays. In contrast, the lack of effect of OM or AM on resting [Ca^2+^]*_i_* came as a surprise, given the ATP depletion observed in galactose- and KIC-cultured cells. It is important to note, however, that the absolute starting ATP concentration within these cells was not known, and a higher resting ATP concentration could explain the inability of OM or AM to induce [Ca^2+^]*_i_* overload, because the relative ATP depletion observed may not have reached the critical ATP threshold required to inhibit the PMCA. Studies aiming to determine the absolute ATP content of cells cultured in glucose-deprived conditions have yielded conflicting results; evidence indicates that cellular ATP can increase following culture in the absence of glucose (37), with cells exhibiting a significantly higher ATP content when the majority of ATP is derived via the mitochondria (25). However, evidence also indicates that galactose-cultured cells have a considerably lower ATP content than glucose-cultured counterparts (21). Nevertheless, regardless of the absolute starting ATP concentration in these cells, the regulation of the PMCA by ATP is complex (38) and can be dynamically regulated by [Mg^2+^]*_i_*, [Ca^2+^]*_i_*, calmodulin, and the phospholipid composition of the plasma membrane (39, 40). Therefore, the absolute ATP threshold at which the PMCA activity is affected in living cells is unknown and likely more complex than originally thought based on cell-free assays.

In addition to its absolute ATP sensitivity, the source and spatial distribution of ATP production (glycolysis *versus* mitochondria) may also impact on the vulnerability of the PMCA to metabolic inhibition. In human erythrocytes, glycolytic enzymes form a complex at the plasma membrane in close proximity to cation pumps (41, 42). Moreover, a supply of glycolytic substrates has been shown to maintain PMCA activity in porcine smooth muscle (43), and this endogenous glycolytic ATP supply was preferentially utilized for [Ca^2+^]*_i_* transport instead of ATP supplemented exogenously (44). It is therefore tempting to speculate that a submembrane glycolytic metabolon may provide a privileged supply of glycolytically derived ATP to the PMCA in PDAC. In this study, a modest but significant decrease in [Ca^2+^]*_i_* clearance rates was observed during the second influx/clearance phase in control KIC and galactose cells. It is likely that ATP is rapidly consumed by the PMCA under the conditions of our *in situ* [Ca^2+^]*_i_* clearance assay, and in light of the aforementioned previous studies, it is tempting to speculate that this decrease in PMCA rate may reflect the lack of a rapid ATP supply from a submembrane glycolytic cascade and the relatively slow diffusion of ATP from mitochondria. Nevertheless, these cells could still recover [Ca^2+^]*_i_*, suggesting that, although rate-limiting for the PMCA, mitochondrial ATP production is sufficient to maintain [Ca^2+^]*_i_* homeostasis.

Taken together, this study supports the notion that glycolytic inhibitors induce PMCA inhibition in PDAC cells due to profound ATP depletion. Moreover, the shift in metabolism observed in galactose- and KIC-cultured cells and their resistance to IAA strongly suggests that PMCA activity and [Ca^2+^]*_i_* homeostasis in PDAC are critically reliant on glycolytic ATP specifically when the glycolytic rate is high. As such, this may be a specific vulnerability of PDAC cells. We propose that the glycolytic ATP supply to the PMCA in PDAC could represent a cancer-specific weakness that could be exploited therapeutically, and it may be an effective strategy for selectively targeting highly glycolytic PDAC tumors.

## Author Contributions

A. D. J., W. P., Z. B., M. A., and R. D. contributed to the experimental work. E. S., A. L., and C. U. contributed to the design of some of the experiments. H. I. developed, validated, and supplied the GO-ATeam FRET reporter. A. K. S. provided clinical insight and contributed to research funding and the overall management of the project. A. D. J. performed most of the experiments and data analysis and generated all figures. A. D. J. and J. I. E. B. designed the experiments, critically evaluated the results, and wrote the manuscript. J. I. E. B. was the principal investigator, obtained funding, and provided the overall academic insight and management of the project.

## Footnotes

5 The abbreviations used are:

PDAC: pancreatic ductal adenocarcinoma
PMCA: plasma membrane Ca^2+^-ATPase
[Ca^2+^]*_i_*: cytosolic Ca^2+^ concentration
HEPES-PSS: HEPES-buffered physiological saline solution
KIC: α-ketoisocaproate
BrPY: 3-bromopyruvate
IAA: sodium iodoacetate
OM: oligomycin
AM: antimycin A
AUC: area under the curve
OCR: O_2_ consumption rate
ECAR: extracellular acidification rate
XF: extracellular flux
AU: absorbance unit
ANOVA: analysis of variance
TRITC: tetramethylrhodamine isothiocyanate.

## References

[1] Amin Z. Theis B. Russell R. C. House C. Novelli M. Lees W. R. (2006) Diagnosing pancreatic cancer: the role of percutaneous biopsy and CT. Clin. Radiol. 61, 996–1002 17097419 10.1016/j.crad.2006.07.005

[2] Tennant D. A. Durán R. V. Gottlieb E. (2010) Targeting metabolic transformation for cancer therapy. Nat. Rev. Cancer 10, 267–277 20300106 10.1038/nrc2817

[3] Le A. Rajeshkumar N. V. Maitra A. Dang C. V. (2012) Conceptual framework for cutting the pancreatic cancer fuel supply. Clin. Cancer Res. 18, 4285–4290 22896695 10.1158/1078-0432.CCR-12-0041 PMC3545437

[4] Chaika N. V. Yu F. Purohit V. Mehla K. Lazenby A. J. DiMaio D. Anderson J. M. Yeh J. J. Johnson K. R. Hollingsworth M. A. Singh P. K. (2012) Differential expression of metabolic genes in tumor and stromal components of primary and metastatic loci in pancreatic adenocarcinoma. PLoS ONE 7, e32996 22412968 10.1371/journal.pone.0032996 PMC3296773

[5] Zhou W. Capello M. Fredolini C. Racanicchi L. Piemonti L. Liotta L. A. Novelli F. Petricoin E. F. (2012) Proteomic analysis reveals Warburg effect and anomalous metabolism of glutamine in pancreatic cancer cells. J. Proteome Res. 11, 554–563 22050456 10.1021/pr2009274 PMC5564318

[6] Cairns R. A. Harris I. S. Mak T. W. (2011) Regulation of cancer cell metabolism. Nat. Rev. Cancer 11, 85–95 21258394 10.1038/nrc2981

[7] Guppy M. (2002) The hypoxic core: a possible answer to the cancer paradox. Biochem. Biophys. Res. Commun. 299, 676–680 12459193 10.1016/s0006-291x(02)02710-9

[8] Guillaumond F. Leca J. Olivares O. Lavaut M.-N. Vidal N. Berthezène P. Dusetti N. J. Loncle C. Calvo E. Turrini O. Iovanna J. L. Tomasini R. Vasseur S. (2013) Strengthened glycolysis under hypoxia supports tumor symbiosis and hexosamine biosynthesis in pancreatic adenocarcinoma. Proc. Natl. Acad. Sci. U.S.A. 110, 3919–3924 23407165 10.1073/pnas.1219555110 PMC3593894

[9] James A. D. Chan A. Erice O. Siriwardena A. K. Bruce J. I. (2013) Glycolytic ATP fuels the plasma membrane calcium pump critical for pancreatic cancer cell survival. J. Biol. Chem. 288, 36007–36019 24158437 10.1074/jbc.M113.502948 PMC3861649

[10] Brini M. Carafoli E. (2009) Calcium pumps in health and disease. Physiol. Rev. 89, 1341–1378 19789383 10.1152/physrev.00032.2008

[11] Marroquin L. D. Hynes J. Dykens J. A. Jamieson J. D. Will Y. (2007) Circumventing the crabtree effect: replacing media glucose with galactose increases susceptibility of HepG2 cells to mitochondrial toxicants. Toxicol. Sci. 97, 539–547 17361016 10.1093/toxsci/kfm052

[12] Bustamante E. Pedersen P. L. (1977) High aerobic glycolysis of rat hepatoma cells in culture: role of mitochondrial hexokinase. Proc. Natl. Acad. Sci. U.S.A. 74, 3735–3739 198801 10.1073/pnas.74.9.3735 PMC431708

[13] Rossignol R. Gilkerson R. Aggeler R. Yamagata K. Remington S. J. Capaldi R. A. (2004) Energy substrate modulates mitochondrial structure and oxidative capacity in cancer cells. Cancer Res. 64, 985–993 14871829 10.1158/0008-5472.can-03-1101

[14] Lenzen S. Schmidt W. Panten U. (1985) Transamination of neutral amino acids and 2-keto acids in pancreatic B-cell mitochondria. J. Biol. Chem. 260, 12629–12634 2864344

[15] Malaisse W. J. Sener A. Malaisse-Legae F. Hutton J. C. Christophe J. (1981) The stimulus-secretion coupling of amino acid-induced insulin release. Metabolic interaction of l-glutamine and 2-ketoisocaproate in pancreatic islets. Biochim. Biophys. Acta 677, 39–49 7028130 10.1016/0304-4165(81)90143-4

[16] Noda C. Ichihara A. (1974) Control of ketogenesis from amino acids. II. Ketone bodies formation from α-ketoisocaproate, the keto-analogue of leucine, by rat liver mitochondria. J. Biochem. 76, 1123–1130 4452666

[17] MacDonald M. J. Fahien L. A. Brown L. J. Hasan N. M. Buss J. D. Kendrick M. A. (2005) Perspective: emerging evidence for signaling roles of mitochondrial anaplerotic products in insulin secretion. Am. J. Physiol. Endocrinol. Metab. 288, E1–E15 15585595 10.1152/ajpendo.00218.2004

[18] Hutton J. C. Sener A. Malaisse W. J. (1979) The metabolism of 4-methyl-2-oxopentanoate in rat pancreatic islets. Biochem. J. 184, 291–301 43143 10.1042/bj1840291 PMC1161764

[19] Shukla S. K. Gebregiworgis T. Purohit V. Chaika N. V. Gunda V. Radhakrishnan P. Mehla K. Pipinos I. I. Powers R. Yu F. Singh P. K. (2014) Metabolic reprogramming induced by ketone bodies diminishes pancreatic cancer cachexia. Cancer Metab. 2, 18 25228990 10.1186/2049-3002-2-18 PMC4165433

[20] Nakano M. Imamura H. Nagai T. Noji H. (2011) Ca^2+^ regulation of mitochondrial ATP synthesis visualized at the single cell level. ACS Chem. Biol. 6, 709–715 21488691 10.1021/cb100313n

[21] Bellance N. Benard G. Furt F. Begueret H. Smolková K. Passerieux E. Delage J. P. Baste J. M. Moreau P. Rossignol R. (2009) Bioenergetics of lung tumors: alteration of mitochondrial biogenesis and respiratory capacity. Int. J. Biochem. Cell Biol. 41, 2566–2577 19712747 10.1016/j.biocel.2009.08.012

[22] Warburg O. (1956) Injuring of respiration the origin of cancer cells. Science 123, 309–314 13298683 10.1126/science.123.3191.309

[23] Frezza C. Gottlieb E. (2009) Mitochondria in cancer: not just innocent bystanders. Semin. Cancer Biol. 19, 4–11 19101633 10.1016/j.semcancer.2008.11.008

[24] Martin M. Beauvoit B. Voisin P. J. Canioni P. Guérin B. Rigoulet M. (1998) Energetic and morphological plasticity of C6 glioma cells grown on 3-D support; effect of transient glutamine deprivation. J. Bioenerg. Biomembr. 30, 565–578 10206476 10.1023/a:1020584517588

[25] Rodríguez-Enríquez S. Gallardo-Pérez J. C. Avilés-Salas A. Marín-Hernández A. Carreño-Fuentes L. Maldonado-Lagunas V. Moreno-Sánchez R. (2008) Energy metabolism transition in multi-cellular human tumor spheroids. J. Cell Physiol. 216, 189–197 18264981 10.1002/jcp.21392

[26] Zu X. L. Guppy M. (2004) Cancer metabolism: facts, fantasy, and fiction. Biochem. Biophys. Res. Commun. 313, 459–465 14697210 10.1016/j.bbrc.2003.11.136

[27] Busk M. Horsman M. R. Kristjansen P. E. van der Kogel A. J. Bussink J. Overgaard J. (2008) Aerobic glycolysis in cancers: implications for the usability of oxygen-responsive genes and fluorodeoxyglucose-PET as markers of tissue hypoxia. Int. J. Cancer 122, 2726–2734 18351643 10.1002/ijc.23449

[28] Nakashima R. A. Paggi M. G. Pedersen P. L. (1984) Contributions of glycolysis and oxidative phosphorylation to adenosine 5′-triphosphate production in AS-30D hepatoma cells. Cancer Res. 44, 5702–5706 6498833

[29] Xu R.-H. Pelicano H. Zhang H. Giles F. J. Keating M. J. Huang P. (2005) Synergistic effect of targeting mTOR by rapamycin and depleting ATP by inhibition of glycolysis in lymphoma and leukemia cells. Leukemia 19, 2153–2158 16193082 10.1038/sj.leu.2403968

[30] Semenza G. L. (2008) Tumor metabolism: cancer cells give and take lactate. J. Clin. Invest. 118, 3835–3837 19033652 10.1172/JCI37373 PMC2582934

[31] Sonveaux P. Végran F. Schroeder T. Wergin M. C. Verrax J. Rabbani Z. N. De Saedeleer C. J. Kennedy K. M. Diepart C. Jordan B. F. Kelley M. J. Gallez B. Wahl M. L. Feron O. Dewhirst M. W. (2008) Targeting lactate-fueled respiration selectively kills hypoxic tumor cells in mice. J. Clin. Invest. 118, 3930–3942 19033663 10.1172/JCI36843 PMC2582933

[32] Gatenby R. A. Gillies R. J. (2004) Why do cancers have high aerobic glycolysis? Nat. Rev. Cancer 4, 891–899 15516961 10.1038/nrc1478

[33] Chen Z. Zhang H. Lu W. Huang P. (2009) Role of mitochondria-associated hexokinase II in cancer cell death induced by 3-bromopyruvate. Biochim. Biophys. Acta 1787, 553–560 19285479 10.1016/j.bbabio.2009.03.003 PMC2731236

[34] Ihrlund L. S. Hernlund E. Khan O. Shoshan M. C. (2008) 3-Bromopyruvate as inhibitor of tumour cell energy metabolism and chemopotentiator of platinum drugs. Mol. Oncol. 2, 94–101 19383331 10.1016/j.molonc.2008.01.003 PMC5527790

[35] Baggaley E. M. Elliott A. C. Bruce J. I. (2008) Oxidant-induced inhibition of the plasma membrane Ca^2+^-ATPase in pancreatic acinar cells: role of the mitochondria. Am. J. Physiol. Cell Physiol. 295, C1247–C1260 18787078 10.1152/ajpcell.00083.2008 PMC2584981

[36] Kim J. S. Ahn K. J. Kim J. A. Kim H. M. Lee J. D. Lee J. M. Kim S. J. Park J. H. (2008) Role of reactive oxygen species-mediated mitochondrial dysregulation in 3-bromopyruvate induced cell death in hepatoma cells: ROS-mediated cell death by 3-BrPA. J. Bioenerg. Biomembr. 40, 607–618 19067133 10.1007/s10863-008-9188-0

[37] Rodríguez-Enríquez S. Vital-González P. A. Flores-Rodríguez F. L. Marín-Hernández A. Ruiz-Azuara L. Moreno-Sánchez R. (2006) Control of cellular proliferation by modulation of oxidative phosphorylation in human and rodent fast-growing tumor cells. Toxicol. Appl. Pharmacol. 215, 208–217 16580038 10.1016/j.taap.2006.02.005

[38] Echarte M. M. Rossi R. C. Rossi J. P. (2007) Phosphorylation of the plasma membrane calcium pump at high ATP concentration. On the mechanism of ATP hydrolysis. Biochemistry 46, 1034–1041 17240987 10.1021/bi061857x

[39] Zhang J. Xiao P. Zhang X. (2009) Phosphatidylserine externalization in caveolae inhibits Ca^2+^ efflux through plasma membrane Ca^2+^-ATPase in ECV304. Cell Calcium 45, 177–184 18929409 10.1016/j.ceca.2008.09.002

[40] Rossi J. P. Rega A. F. (1989) A study to see whether phosphatidylserine, partial proteolysis and EGTA substitute for calmodulin during activation of the Ca^2+^-ATPase from red cell membranes by ATP. Biochim. Biophys. Acta 996, 153–159 2526658 10.1016/0167-4838(89)90241-0

[41] Puchulu-Campanella E. Chu H. Anstee D. J. Galan J. A. Tao W. A. Low P. S. (2013) Identification of the components of a glycolytic enzyme metabolon on the human red blood cell membrane. J. Biol. Chem. 288, 848–858 23150667 10.1074/jbc.M112.428573 PMC3543034

[42] Campanella M. E. Chu H. Low P. S. (2005) Assembly and regulation of a glycolytic enzyme complex on the human erythrocyte membrane. Proc. Natl. Acad. Sci. U.S.A. 102, 2402–2407 15701694 10.1073/pnas.0409741102 PMC549020

[43] Paul R. J. Hardin C. D. Raeymaekers L. Wuytack F. Casteels R. (1989) Preferential support of Ca^2+^ uptake in smooth muscle plasma membrane vesicles by an endogenous glycolytic cascade. FASEB J. 3, 2298–2301 2528493 10.1096/fasebj.3.11.2528493

[44] Hardin C. D. Raeymaekers L. Paul R. J. (1992) Comparison of endogenous and exogenous sources of ATP in fueling Ca^2+^ uptake in smooth muscle plasma membrane vesicles. J. Gen. Physiol. 99, 21–40 1311020 10.1085/jgp.99.1.21 PMC2216599

